# Economic analysis of a new four-panel rapid screening test in antenatal care in Kenya, Rwanda, and Uganda

**DOI:** 10.1186/s12913-023-09775-z

**Published:** 2023-07-31

**Authors:** Donald S Shepard, Yara A Halasa-Rappel, Katharine R Rowlands, Maria Kulchyckyj, Robert K Basaza, Emmanuel D Otieno, Boniface Mutatina, Simon Kariuki, Sabine F Musange

**Affiliations:** 1 The Heller School for Social Policy & Management, Brandeis University, Waltham, MA 02454-9110 USA; 2 Commonwealth Medicine, University of Massachusetts Medical School, Worcester, MA USA; 3 Uganda Christian University, Mukono, Uganda; 4 Bugema University, Bugema, Uganda; 5 Makerere University School of Medicine, Kampala, Uganda; 6 Kenya Medical Research Institute, Kisian, Kenya; 7 University of Rwanda, Kigali, Rwanda

**Keywords:** Cost-effectiveness, Antenatal care, Hepatitis B, HIV/AIDS, Malaria, Syphilis, Kenya, Rwanda, Uganda

## Abstract

**Background:**

We performed an economic analysis of a new technology used in antenatal care (ANC) clinics, the ANC panel. Introduced in 2019–2020 in five Rwandan districts, the ANC panel screens for four infections [hepatitis B virus (HBV), human immunodeficiency virus (HIV), malaria, and syphilis] using blood from a single fingerstick. It increases the scope and sensitivity of screening over conventional testing.

**Methods:**

We developed and applied an Excel-based economic and epidemiologic model to perform cost-effectiveness and cost-benefit analyses of this technology in Kenya, Rwanda, and Uganda. Costs include the ANC panel itself, its administration, and follow-up treatment. Effectiveness models predicted impacts on maternal and infant mortality and other outcomes. Key parameters are the baseline prevalence of each infection and the effectiveness of early treatment using observations from the Rwanda pilot, national and international literature, and expert opinion. For each parameter, we found the best estimate (with 95% confidence bound).

**Results:**

The ANC panel averted 92 (69–115) disability-adjusted life years (DALYs) per 1,000 pregnant women in ANC in Kenya, 54 (52–57) in Rwanda, and 258 (156–360) in Uganda. Net healthcare costs per woman ranged from $0.53 ($0.02-$4.21) in Kenya, $1.77 ($1.23-$5.60) in Rwanda, and negative $5.01 (-$6.45 to $0.48) in Uganda. Incremental cost-effectiveness ratios (ICERs) in dollars per DALY averted were $5.76 (-$3.50-$11.13) in Kenya, $32.62 ($17.54-$46.70) in Rwanda, and negative $19.40 (-$24.18 to -$15.42) in Uganda. Benefit-cost ratios were $17.48 ($15.90-$23.71) in Kenya, $6.20 ($5.91-$6.45) in Rwanda, and $25.36 ($16.88-$33.14) in Uganda. All results appear very favorable and cost-saving in Uganda.

**Conclusion:**

Though subject to uncertainty, even our lowest estimates were still favorable. By combining field data and literature, the ANC model could be applied to other countries.

**Supplementary Information:**

The online version contains supplementary material available at 10.1186/s12913-023-09775-z.

## Introduction

In 2019, the estimated prevalence of hepatitis B virus (HBV), human immunodeficiency virus (HIV), syphilis, and malaria among women of reproductive health age (15–49) in sub-Saharan Africa was 244, 4,717, 2,813, and 11,753 per 100,000 women of reproductive health age, respectively [1]. Antenatal care (ANC) clinics are well-positioned to provide a platform for essential healthcare functions, including screening pregnant women for infectious diseases.

### Hepatitis B virus

HBV is a viral infection of the liver transmitted through contact with an infected individual’s bodily fluids or blood. Globally, HBV vaccination programs reduced the incidence of acute hepatitis B and the prevalence of chronic hepatitis B surface antigen (HBsAg) carriers. However, HBV infection remains highly endemic in sub-Sahara Africa, with an estimated incidence of 0.30% (confidence bound: 0.24-0.37%) [2] or 65 million chronic HBsAg carriers, of whom 25% are expected to die from liver disease, including cirrhosis, hepatocellular carcinoma, and liver failure [3, 4]. Little is known about the natural history of chronic HBV infection during pregnancy or its impact on pregnancy outcomes [5, 6]. However, universal prenatal screening for HBV, by testing for HBsAg, is recommended for all pregnant women during their first ANC visit to reduce perinatal transmission of HBV and the subsequent development of chronic HBV infection [7]. Screening has a low false-positive rate, and treatment is rarely harmful [8, 9].

For pregnant women who test positive for HBsAg, antiviral treatment with tenofovir disoproxil fumarate (TDF) is recommended during the last trimester. The treatment could be discontinued after delivery or 3 months postpartum [10]. To prevent perinatal transmission of HBV, infants must receive hepatitis B vaccine with hepatitis B immunoglobulin (HBIg) prophylaxis treatment within 24 h of birth and complete the HBV vaccination series within 18 months [11, 12]. Without treatment, acute HBV infections progress to chronic disease in 80–90% of infected infants [3]. Chronic HBV infection increasess the long-term morbidity and mortality of infected children and the chance of developing cirrhosis of the liver and liver cancer. Approximately 25% of persons who become chronically infected during childhood and 15% of those infected as adults will die of cirrhosis or hepatocellular carcinoma [13].

### HIV

HIV is a virus that attacks the body’s immune system. Untreated, the patient would develop acquired immunodeficiency syndrome (AIDS) [14]. Biological factors associated with pregnancy increase female susceptibility to HIV acquisition. The high levels of estrogen and progesterone that accompany pregnancy can induce a cascade of synergistic changes within the female genital tract, increasing inflammation, decreasing the integrity of the vaginal epithelium, and causing alterations in vaginal microbiota, all of which have been associated with increased HIV acquisition susceptibility. Pregnancy activates innate immunity, increasing inflammation and HIV target cells in the female genital tract while simultaneously suppressing adaptive immunity and reducing natural killer cells, changes that can persist as long as nine months after delivery [15]. In sub-Saharan Africa, the prevalence of HIV/AIDs comprises 2.28% (confidence bound: 1.92-2.70%) of the population [2], with adolescent girls and young women twice as likely to be infected compared to boys and men of the same age [16]. HIV testing and linkage to care, screening and treatment of sexually transmitted diseases, condom promotion, and partner engagement strategies are recommended to prevent HIV during pregnancy [17]. For pregnant women living with HIV, perinatal transmission of HIV could occur during pregnancy, childbirth, or breastfeeding [18]. In the absence of treatment, the risk of perinatal transmission of HIV among non-breastfeeding mothers is 15-30% and increases to 20-45% among breastfeeding mothers [19]. In sub-Saharan Africa, the rate of perinatal transmission of HIV is still high, accounting for nearly 90% of the 1.8 million children living with HIV. With the implementation of the World Health Organization’s (WHO) 2010 recommendation to use antiretroviral treatment (ART) drugs for controlling and preventing HIV infection [19], ART coverage to prevent perinatal transmission of HIV increased from 32.98% in 2010 to 69.46% in 2019 for HIV-positive pregnant women; as a result, the rate of mother-to-child HIV transmissions fell from 27.18 to 16.90 per 100 live births [20]. However, in 2019, the average ART coverage in sub-Saharan Africa varied by country, ranging from 4% in Sudan, 78% in Kenya to 100% in Benin, Botswana, Malawi, Mozambique, Namibia, and Uganda, and 95% in Rwanda [20, 21]. For countries with a high burden of HIV, the WHO recommends using a rapid diagnostic test (RDT) and/or enzyme immunoassay combination to achieve a 99% positive predictive value (to ensure the probability of an HIV-positive test being correct) [22]. Daily oral Truvada™ is approved for HIV pre-exposure prophylaxis (PrEP) in all populations at risk and is safe and effective in pregnant and breastfeeding women at risk of HIV infection [17], and various ART regimens are safe and effective in preventing HIV infection in infants [19].

### Syphilis

Syphilis is a sexually transmitted disease caused by *Treponema pallidum*, which can also be transmitted through blood transfusion and perinatal transmission. Mother-to-child transmission of syphilis has harmful adverse outcomes for the fetus if the maternal infection is not detected and treated early [23]. In 2016, there were 1.1 million cases of maternal syphilis globally, resulting in more than 660,000 cases of congenital syphilis, of which 350,000 occurred as an adverse birth outcome, with the majority of these cases in sub-Saharan Africa and Asia [23–25]. Most syphilis cases are asymptomatic. Syphilis could be diagnosed using a serological test, either non-treponemal or treponemal. A presumptive diagnosis of syphilis requires a positive result from at least one of those tests. A confirmed diagnosis requires positive results from both types of serologic tests [23]. Rapid syphilis tests offer screening for syphilis at point-of-care and provide treponemal antibody results within 10–15 minutes. However, a positive test indicates a presumptive diagnosis, and a confirmed test is recommended [26, 27]. Syphilis is treated with penicillin, which is safe and effective during pregnancy, primarily during the first trimester. Penicillin is available at Second Generation of Health Posts and all higher-level facilities. Appropriate treatment of syphilis prevents congenital syphilis and significantly decreases adverse pregnancy outcomes. Left untreated, syphilis in pregnancy could lead to adverse health outcomes, including stillbirth, preterm birth, and neonatal asphyxia [28, 29].

### Malaria

Malaria in pregnancy is a public health challenge. An estimated 125 million pregnant women reside in endemic areas, putting them at high risk of contracting malaria, especially in sub-Saharan Africa, where the incidence of malaria is estimated at 2.12% annually (confidence bound 1.51-2.84%) among women between 15 and 49 years of age, and 12–20% of stillbirths are attributed to malaria [2, 30]. The clinical effects of malaria on pregnant women vary from no symptoms to severe anemia and death. Malaria is diagnosed using RDTs such as *Plasmodium falciparum* histidine-rich protein-2 (PfHRP2). Currently, intermittent prophylactic therapy in pregnancy (IPTp) is recommended only in Africa, using sulphadoxine-pyrimethamine (SP). A meta-analysis showed that IPTp remains effective against low birth weight and anemia, even when resistance to SP is high, which is common in eastern and southern Africa [30].

Implementing timely and evidence-based practices, including screening, during ANC visits could save lives [31]. Most congenital infections result from asymptomatic or symptomatic maternal infections [32]. However, in resource-scarce countries, the need for multiple tests and shortages of personnel and test kits might hinder the ability of healthcare providers to screen, diagnose, and treat pregnant women in a timely manner. Abbott Laboratories developed a new ANC panel rapid diagnostic test that screens for four infections (HBV, HIV, syphilis, and malaria) using a blood sample from a single fingerstick and increased the scope and sensitivity of screening. This ANC panel was piloted in five districts in Rwanda between 2019 and 2020. This report estimates the economic benefits of the new ANC panel rapid diagnostic test in three countries in sub-Saharan Africa: Kenya, Rwanda, and Uganda, using societal and healthcare system perspectives. We hypothesize that the new ANC panel will complement the current diagnostic system used in those countries.

## Methods

### Model framework

**Fig. 1:**
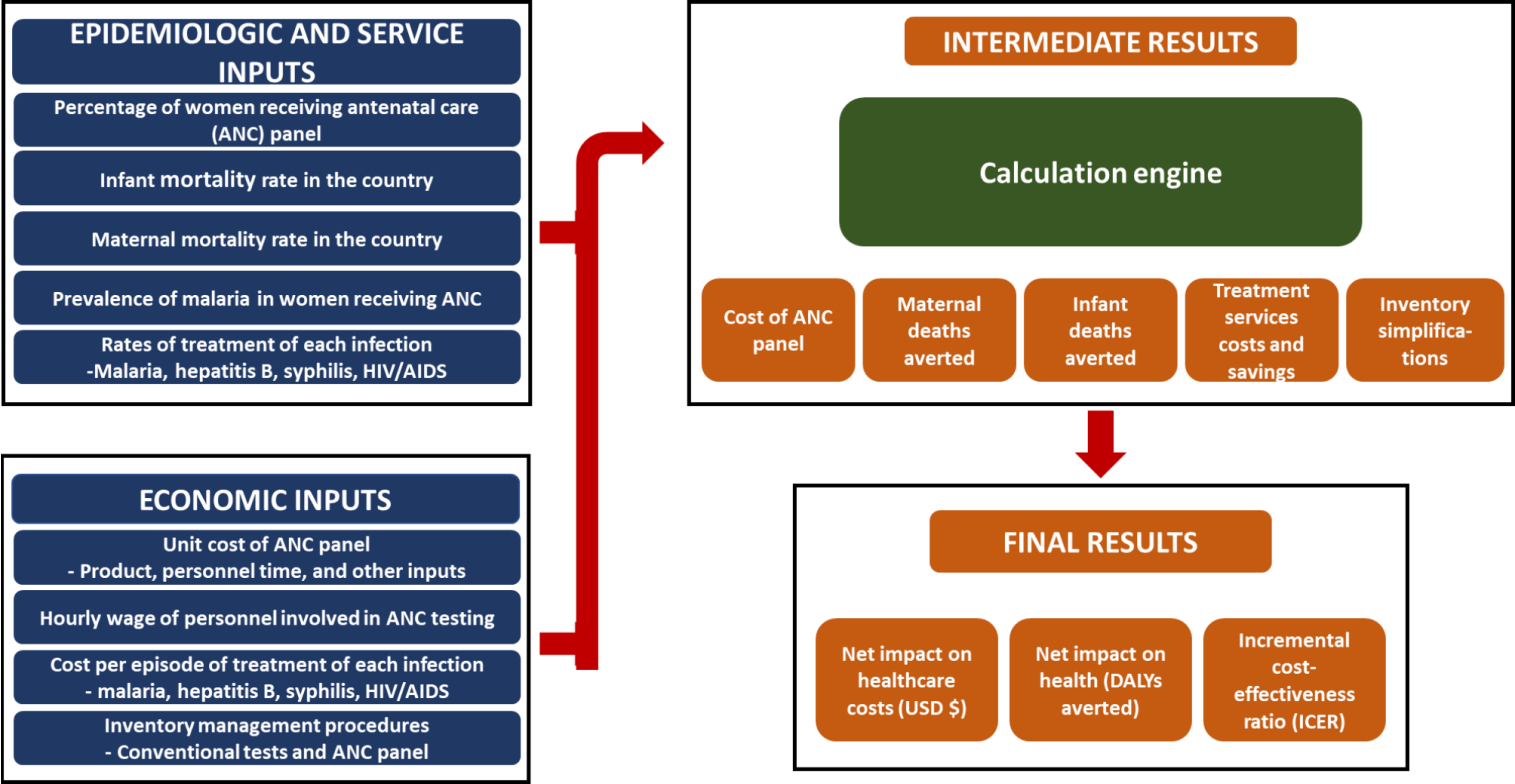
The ANC panel economic model flow chart Notes: ANC denotes antenatal care; ICER denotes incremental cost-effectiveness ratio; USD denotes United States dollars

We used an economic evaluation framework to estimate the incremental benefits of introducing the ANC panel [33]. As highlighted in Fig. 1, the benefits are associated with the incremental increase in pregnant women attending ANC services screened for HBV, HIV, syphilis, and malaria. The net cost of introducing the ANC panel combines expenses on the ANC panel, its administration, and follow-up treatment compared to conventional practices. To determine the effectiveness, we reviewed the literature on the impacts of each of the four infections on maternal and infant health. We then modeled the effect of each maternal infection as the incremental change in the disability-adjusted life years (DALYs) averted due to the ANC panel. DALYs averted were estimated as a function of the maternal and infant mortality rate, life expectancy, positivity rate of each infection, and rates of treatment successes attributed to the introduction of the ANC panel. We developed the ANC panel economic model as a customized Excel workbook automating and linking calculations. We used this model to estimate the value of introducing the ANC panel in Kenya, Rwanda, and Uganda by estimating the cost and effectiveness of the ANC panel for a hypothetical cohort of 1,000 women receiving ANC.

Our framework assumes that women are routinely tested only once durning each pregnancy, generally at their first ANC visit. Those testing positive are offered treatment specific to the infection found. For example, the diagnosis of syphilis is based on clinical history, physical examination, laboratory testing, and sometimes radiology. The syphilis component of the ANC panel is the Determine™ Syphilis TP test. It detects antibodies to *Treponema pallidum*, the bacteria that causes syphilis, but does not inform health providers if the disease is active [34]. All women who test positive on this test are given benzathine penicillin, entailing one additional visit plus the cost of the penicillin. This treatment is inexpensive and safe (with minimal adverse effects). Based on the cost of the visit, drugs, and supplies, we estimated the cost at $2.52 per woman in 2020 prices, combining the visit, drugs, and supplies.

### Overall data sources

We calibrated the model from peer-reviewed literature, facility reports from the Rwanda pilot, websites of the Institute of Health Metrics and Evaluation (IHME), World Health Organization (WHO), United Nations AIDS Organization (UNAIDS) data observatory, and national reports. We supplemented gaps in the literature with expert judgment. Table 1 and the text below present the epidemiological data collected for each country and the source of information. Table 2 presents the cost of testing and treatment used for each country.

**Table 1:** The ANC panel economic model epidemiological inputs for Kenya, Rwanda, and Uganda

Indicator	Kenya	Rwanda	Uganda
Infant mortality rate, per 1,000 live births	31.9 [35]	33.0 [36]	33.4 [37]
Maternal mortality rate, per 100,000 live births	342 [38]	203 [36]	336 [39]
Life expectancy at birth, years	66.7 [40]	67.7 [41]	63.0 [42]
Average maternal age at delivery, years	20 [43]	23 [44]	25 [45]
Testing rate using conventional tests in ANC settings			
Testing rate for HBV	97.7% [46]	0.0%^a^	0.0%^a^
Testing rate for HIV	88.9% [47]	86.2%^b^	99.0% [48]
Testing rate for syphilis	98.6% [49]	85.0%^b^	87.0% [50]
Testing rate for malaria	20.8%^c^ [51–53]	25.0%^d^	16.0%[54]
Positivity rate among pregnant women			
Positivity rate for HBV	9.30% [46]	3.90% [55]^e^	4.40% [56]
Positivity rate for HIV	6.00% [47]	0.65%^f^	1.70% [57, 58]
Positivity rate for syphilis	1.24% [49]	8.44%^f^	3.00% [56]
Positivity rate for malaria	31.90% [52]	2.58%^f^	16.00% [54]

Notes: ANC denotes antenatal care; HBV denotes hepatitis B virus; HIV denotes human immunodeficiency viruses

^a^ Based on our understanding of current practices in the country. Public health authorities in Bugesera District reported no routine testing prior to the initiation of the ANC panel

^b^ Based on the abstraction of data from registers of health centers serving control and intervention cells in Bugesera District

^c^ Based on coverage in the private sector, ACT Watch Group [53]. and Young, et al. [52] found no data on malaria in the public sector, but that does not mean testing does not exist, so we took the average of testing reported in Rwanda and Uganda

^d^ Based on fact that malaria testing and treatment are offered in outpatient clinics but not connected to ANC care

^e^ The reported prevalence of hepatitis B by Makuza et al. [55] is the best estimate available since it is the most recent, covers a large part of Rwanda, and the credibility of the results was enhanced by a prior paper that found the prevalence among HIV population to be 3.7% [59]

^f^ Monica Sanders, Abbot, Inc., ANC panel positivity report (personal communication, received March 22, 2021)

**Table 2:** The ANC panel economic model cost inputs for Kenya, Rwanda, and Uganda, in 2020

Indicator	Kenya	Rwanda	Uganda
Cost of ANC panel (from Abbott, Inc.)	$4.00	$4.00	$4.00
Hepatitis B test unit cost	$3.99^a^	$2.69	$1.40^b^
HIV test unit cost	$1.48	$1.35 [60]	$1.40 [60]
Malaria test unit cost	$0.50 [60]	$0.58	$0.50 [61]
Syphilis test unit cost	$1.50 [60]	$0.82	$3.00 [62]
Hepatitis B treatment: consultation fees 1 visit per month x 24 months^c^	$94.56 [63]	$56.96	$58.72^d^
Hepatitis B medication: tenofovir 300 mg 1x/day x 24 months [60]	$29.92	$29.92	$29.92
Hepatitis B vaccine for infants, 2-doses ($0.25 per dose) [64]	$0.50	$0.50	$0.50
Average cost of treatment for chronic hepatitis B infection (consultation + treatment + infant vaccination	$399.06	$38.71	$89.14
Average cost of PMTCT per mother-baby pair (2 years of treatment)	$486 [65]	$447.29 [66]	$441.90 [66]
Malaria treatment: consultation fee per visit	$11.82 [63]	$1.04	$4.00 [67]
Malaria medication: Coartem (1 blister pack)	$0.27 [68, 69]	$0.73	$1.70 [70]
Average cost of malaria episode (consultation + treatment)	$12.09	$1.77	$7.58 [71]
Syphilis treatment: consultation fee per visit	$11.82 [63]	$1.04	$4.00 [67]
Syphilis medication: treatment for one infection not including consultation fee	$2.50 [72]	$0.75	$0.76
Hourly wage of a nurse	$3.18	$0.86^e^	$0.81^e^
Hourly wage of laboratory technician	$3.88	$0.55^e^	$0.52^e^
Cost of healthy pregnancy outcome	$82.47 [73]	$82.47	$104.00 [74]
Cost of neonatal death [73]	$82.47	$82.47	$82.47
Cost of congenital syphilis [73]	$914.10	$914.10	$914.10
Cost of low birthweight outcome [73]	$1,714.31	$1,714.31	$1,714.31
Cost of lifetime care for HIV-positive infant	$698 [75]	$447.29 [66]	$675.90^f^

Notes: ANC denotes antenatal care; PMTCT denotes prevention of mother to child transmission. Where not otherwise indicated, costs for Rwanda are based on the country’s reimbursement rates for public insurance

^a^KEMRI, Centre for infectious and parasitic diseases control centre [76].

^b^ Expert opinion

^c^Adjusted to accommodate the authors’ assumption that patients will adhere one month every 3 months

^d^ Consultation fees ($7.34) 1 visit per month x 24 months. IHME, ABCE report for treatment adherence [77]; Uganda 2014 (Adjusted for expert judgment: patients come one month out of three); Derived from cost for uninsured in Rwanda

^e^ Interviews with staff at 4 Second Generation of Health Posts

^f^ Authors’ estimate based on the country’s Gross National Income

Based on the pilot study in Rwanda, we estimated the cost of administering the ANC panel in the three countries included in this study. The Rwanda cost includes the personnel costs (a nurse’s average hourly wage of $0.86, and a laboratory technician’s hourly rate of $0.55) and the cost of the 4-test ANC panel ($4.00), which was provided by the manufacturer. Supplemental Table S1 presents the average time spent on testing by diagnostic test type.

The current rate of treatments for the four infections and other information to which national representative data were not available, we relied on our epidemiological country co-authors for expert judgment. In Kenya, our country co-author (SK) is a senior official at the Global Health section at Kenya Medical Research Institute. In Rwanda, our country co-author (SFM) is a lecturer at the School of Public Health, National University of Rwanda and an advisor to the country’s Ministry of Health. In Uganda, our country expert (RKB) is a faculty member at Bugema University and a formal official of the Uganda Ministry of Health.

### Kenya sources

In Kenya, we estimated a hepatitis B infection rate (with a sensitivity analysis from least to most favorable) of 38 (19–57), an HIV infection rate of 60 (30–90), a malaria infection rate of 319 (159.5-478.5), and syphilis infection rate of 12.4 (6.2–18.6) per 1,000 pregnant women. Based on conventional test coverage, we estimated the incremental increase in testing rate due to the ANC panel to be 99% for HBV, 0.65% for HIV, 9.4% for malaria, and 14.4% for syphilis. To estimate the effectiveness of the ANC panel from the literature, we derived a 0.0097% case fertility rate for pregnant women due to HBV infection [55, 78] 0.138% due to HIV [78, 79], and 0.724% due to malaria infection [78, 80]. In addition, we derived a 17.5% infant mortality rate due to HIV as a result of perinatal transmission [2], 3.6% due to malaria [50], and 6.0% due to syphilis [81]. From the IHME global burden of disease (GBD) database [2], we used 0.0262 as the value of DALYs lost per person per year due to HBV, 0.334 for HIV, 0.223 for malaria, and 1.09 for syphilis.

### Rwanda sources

In Rwanda, we estimated a hepatitis B infection rate of 39.0 (38.3–39.7), an HIV infection rate of 26 (13–39), a malaria infection rate of 19 (10–29), and syphilis infection rate of 20 (10–30) per 1,000 pregnant women. Based on conventional test coverage, we estimated the incremental increase in testing rate due to the ANC panel to be 99.4% for HBV, 0.65% for HIV, 9.4% for malaria, and 14.4% for syphilis.

### Uganda sources

In Uganda, we estimated the following infection rates per 1,000 pregnant women: HBV 44 (22–66), HIV 17.0 (8.5–25.5), malaria 160 (80–240) and syphilis 30 (15–45). Based on conventional test coverage, we estimated the incremental increase in testing rate due to the ANC panel to be 99% for HBV, 0.35% for HIV, 83.4% for malaria, and 12.4% for syphilis. The current rates of treatment for the four infections were obtained from nationally representative data where possible; when not available, we relied on our epidemiological country co-authors for expert judgment.

### Analytical approaches

To estimate the number of maternal and infant deaths averted, we computed the share of new infections captured due to the introduction of the ANC panel and multiplied it by the maternal and infant case fatality rate. We estimated the incremental maternal and infant infections detected and treated per 1,000 pregnant women due to the introduction of the ANC panel and the incremental maternal and infant DALYs per 1,000 births averted using the ANC panel. DALYs averted were discounted using a 3% annual discount rate.

For the cost-effectiveness analysis, we considered the following costs for this analysis using country-specific epidemiological and cost of illness literature review, charges paid by uninsured individuals for conventional tests, and the results of an ANC pilot conducted at Bugesera District in Rwanda in 2019: The gross cost of the ANC panel includes (1) the gross cost of test supplies, (2) the gross incremental cost of treating the four infections, and (3) the gross personnel cost of administering the ANC panel. For the economic evaluation under the health system perspective, we included the costs of the ANC panel, as described above, plus medical costs averted from early diagnosis and treatment. For the societal perspective in the benefit-cost analysis, we used all the elements from the cost-effectiveness analysis and incorporated the economic value of DALYs averted. We computed it as the product of DALYs averted by the country’s gross per capita national income (GNI).

For the cost-effectiveness analysis, we estimated the net cost of the ANC panel as the difference between the ANC panel’s gross cost and saving from (1) fewer adverse pregnancy outcomes, (2) infant healthcare costs averted, (3) test supplies from conventional tests averted, and (4) personnel wages from conventional tests averted. We estimated the incremental cost-effectiveness ratio (ICER) as the net cost of the ANC panel divided by the net DALYs averted due to the ANC panel.

For the benefit-cost analyses, we categorized the gross cost of the ANC panel into its the monetary benefit, which includes: cost-saving from fewer adverse pregnancy outcomes, the economic value of DALYs averted, cost-saving from administering conventional tests, and cost-saving in the healthcare system (e.g., personnel time). The gross benefits from the societal perspective include the economic value of DALYs. We computed the healthcare cost offsets from the health system perspective as the cost-saving from fewer adverse pregnancy outcomes, cost-saving from not administering some conventional tests, and cost-saving in the healthcare system, excluding the economic cost of DALYs averted.

### Sensitivity analyses

We conducted a probabilistic sensitivity analysis. To implement this, we adopted a three-valued discrete probability distribution for the incidence or prevalence of each disease. Lacking further information about the detailed distribution, each of these three values served as a proxy for surrounding values. We assigned a probability of 0.10 to its lower 95% confidence bound, a 0.80 probability to the best or central estimate, and a 0.10 probability to the upper 95% confidence bound. We assumed that the four diseases were statistically independent. This process generated a discrete distribution with 81 values. The probability attached to each value was the product of probabilities for each disease. As impacts on costs and DALYs averted summed across the diseases, we computed each disease’s impact based on its deviation from the best estimate. By calculating cumulative probabilities, we calculated the 95% confidence bound for the ICER and the benefit-cost ratio.

## Results

### Net costs

Table 3 presents the economic evaluation of the ANC panel from a health system perspective. The net cost to the health care system associated with the introduction of the ANC panel varies by country. The ANC panel was associated with marginal additional costs (with confidence bound) per pregnant woman visiting ANC clinics of $0.53 ($0.02-$4.21) in Kenya and $1.77 ($1.23-$5.60) in Rwanda, and cost savings of $5.01 (-$6.45-$0.48) in Uganda. The ANC panel averted 92 (68.98-114.71) DALYs per 1,000 pregnant women in ANC in Kenya, 54.32 (51.93–56.56) in Rwanda, and 258.29 (156.35-359.62) in Uganda.

**Table 3:** The cost and benefits of introducing the ANC panel per 1,000 pregnant women

Item	Lower 95% CB	Best estimate	Upper 95% CB
Kenya			
Net healthcare cost	-$245.17	$530.00	$1,272.83
Net health gains (DALYs averted)	68.98	92.00	114.71
Incremental cost-effectiveness ratio (ICER)	-$3.50	$5.76	$11.13
*Net healthcare cost per pregnant woman*	$0.02	$0.53	$4.21
Rwanda			
Net healthcare cost	$920	$1,772	$2,599
Net health gains (DALYs averted)	51.93	54.32	56.56
Incremental cost-effectiveness ratio (ICER)	$17.54	$32.62	$46.70
*Net healthcare cost per pregnant woman*	$1.23	$1.77	$5.60
Uganda			
Net healthcare cost*	-$7,525.92	-$5,009.84	-$2,516.08
Net health gains (DALYs averted)	156.35	258.29	359.62
Incremental cost-effectiveness ratio (ICER)*	-$24.18	-$19.40	-$15.42
Net healthcare cost per pregnant woman*	-$6.45	-$5.01	$0.48

Notes: * negative $ sign indicates cost saving. ANC denotes antenatal care; CB denotes confidence bound; DALYs denotes disability-adjusted life years; ICER denotes incremental cost-effectiveness ratio

### Cost-effectiveness results

**Fig. 2:**
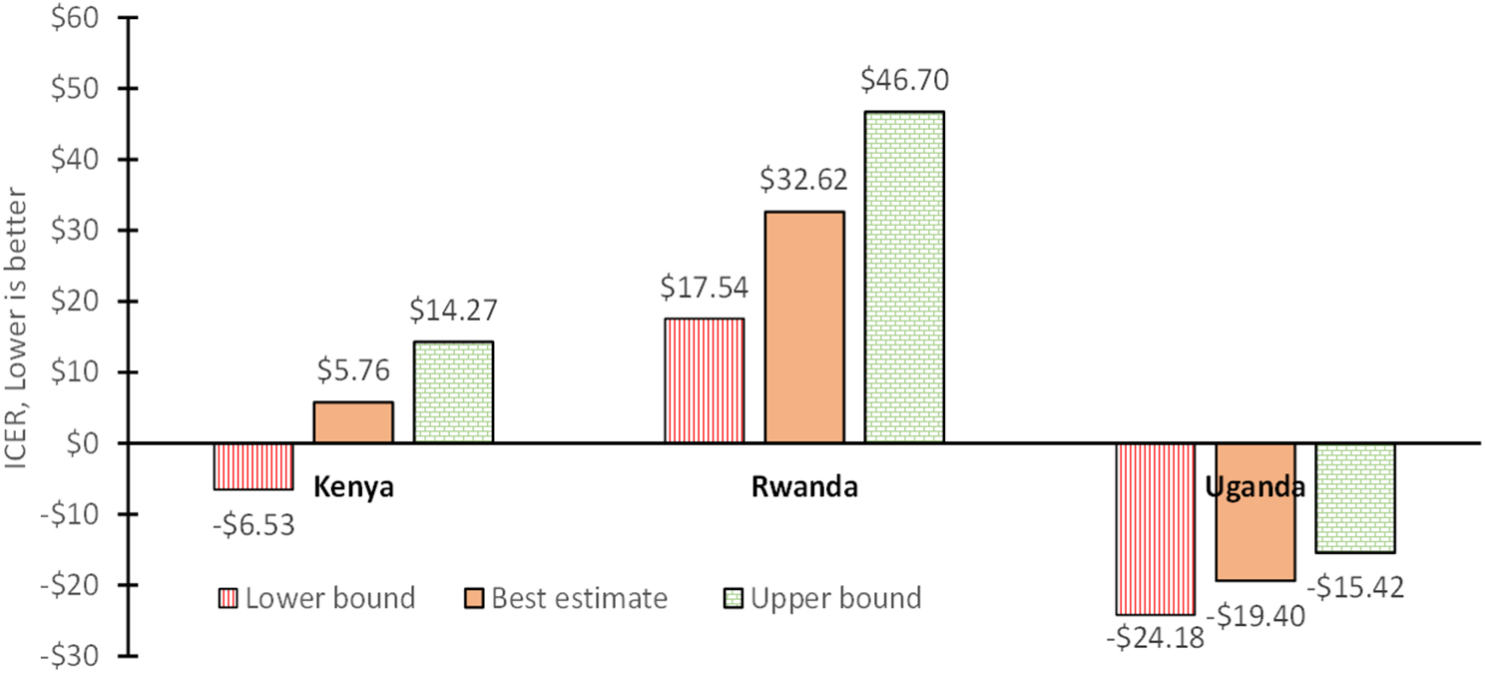
Incremental cost-effectiveness ratio (ICER), $/DALY Notes: DALY denotes disability-adjusted life years; ICER denotes incremental cost-effectiveness ratio. Bounds are lower and upper 95% confidence bounds

Figure 2 shows the incremental cost-effectiveness ratio of introducing the ANC panel in each country. In Kenya, the introduction of the ANC panel was associated with a cost of $5.76 (-$6.53-$14.27) per DALY averted, well under the country’s $1,759 GNI per capita, making the ANC panel a highly cost-effective intervention. In Rwanda, the introduction of the ANC panel was associated with a cost of $32.62($17.54-$46.70) per DALY averted, also well under the country’s $830 GNI per capita, again making the ANC panel a highly cost-effective intervention (see Supplemental material S2). In Uganda, it was associated with a saving of $19.40 (-$24.18–$15.42) per DALY averted, making the intervention cost saving— even more favorable than highly cost-effective.

### Benefit-cost results

**Fig. 3:**
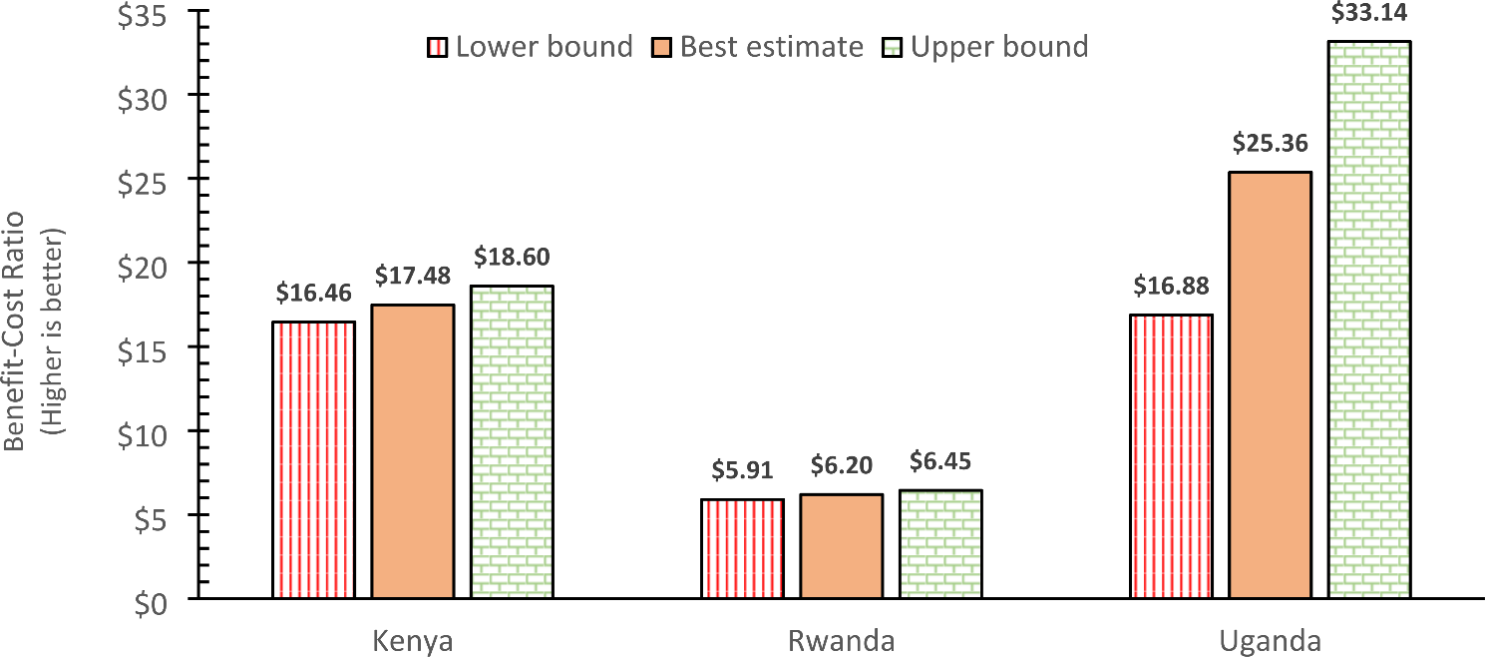
Benefit-cost ratio ($ benefits per dollar invested) of introducing the ANC panel Note: Bounds are lower and upper 95% confidence bounds. ANC denotes antenatal care

The benefit-cost results found that each $1 spent on the ANC panel is associated with economic benefits of $17.48 ($16.46-$18.60) in Kenya, $6.20 ($5.91-$6.45) in Rwanda, and $25.36 ($16.88-$33.14) in Uganda (see Fig. 3). Most of the economic benefits are due to the value of DALYs averted per 1,000 pregnant women: $ 161,035 ($121,873-$198,485) in Kenya, $42,367 ($22,218-$62,355) in Rwanda, and $214,380 ($133,955-$294,294) in Uganda (Table 4).

**Table 4:** Monetary benefits and costs of introducing the ANC panel per 1,000 pregnant women

Indicator	Lower 95% CB	Best estimate	Upper 95% CB
Kenya			
Overall benefits	$130,205	$170,243	$210,019
Benefits from DALYs saved	$121,873	$161,035	$198,485
Benefits from saving to the healthcare system	$8,332	$9,208	$11,534
Gross costs	$7,910	$9,739	$11,291
Rwanda			
Overall benefits	$25,429	$48,402	$71,284
Benefits from DALYs saved	$22,218	$42,367	$62,355
Benefits from saving to the healthcare system	$3,212	$6,035	$8,929
Gross costs	$4,303	$7,807	$11,052
Uganda			
Overall benefits	$145,325	$228,395	$310,949
Benefits from DALYs saved	$133,955	$214,380	$294,294
Benefits from saving to the healthcare system	$11,370	$14,014	$16,655
Gross costs	$8,609	$9,006	$9,383

Notes: ANC denotes antenatal care; CB denotes confidence bound; DALYs denotes disability-adjusted life years

**Fig. 4:**
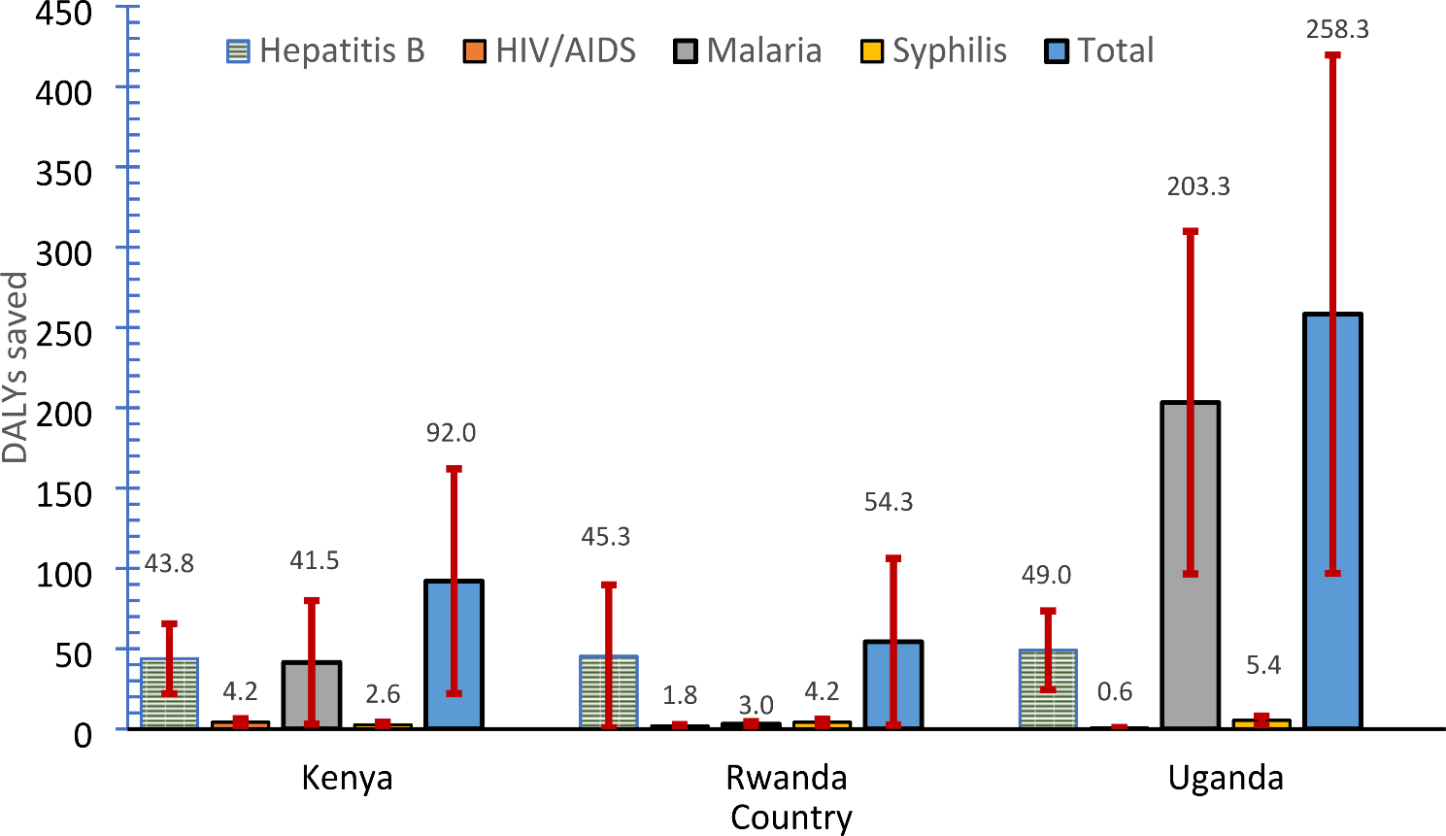
Additional DALYs saved by ANC panel per 1,000 pregnant women by country and infection (with confidence bound) Notes: ANC denotes antenatal care; DALYs denotes disability-adjusted life years. Bars denote lower and upper 95% confidence bound

As presented in Fig. 4 and Supplemental Table S3, the majority (92.6%) of DALYs averted in Kenya was due to early detection and treatment of hepatitis B and malaria. In Rwanda, 83.4% of DALYs averted were due to early detection and treatment of hepatitis B, followed by syphilis (7.7%). In Uganda, 78.7% of the DALYs averted were due to malaria, and 19.0% for early detection and treatment of hepatitis B.

### Sensitivity analysis results

**Fig. 5:**
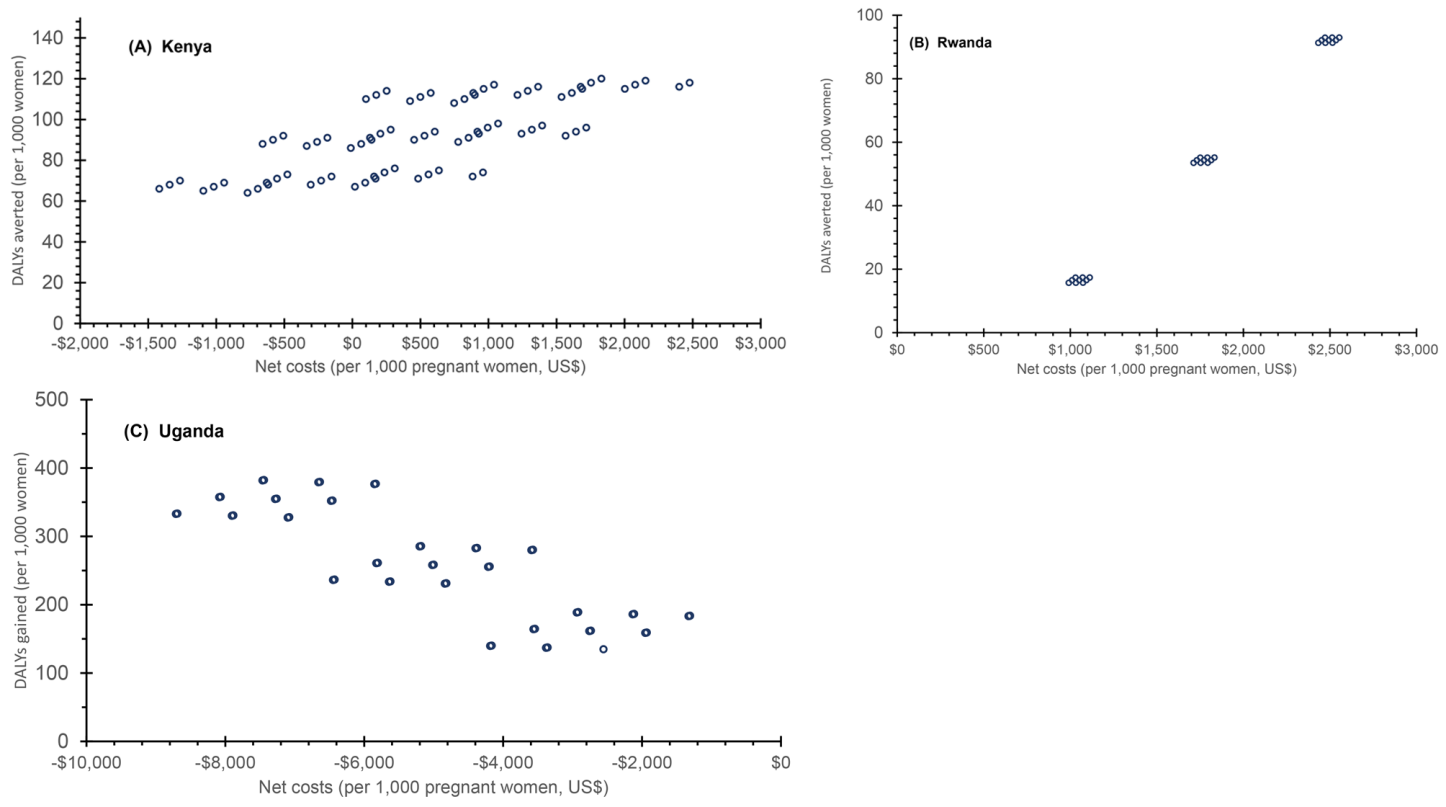
Scatter plots of net costs and net effectiveness by country Note: Each of the 81 points in the scatter plot for each country represents a unique combination of the incidence of the four infections (each with three possibilities) in that country, i.e., 3 × 3 × 3 × 3 = 81. In some cases, the results overlap, so the separate points are not distinguishable. DALYs denotes disability-adjusted life years

Figure 5 shows the probabilistic sensitivity analyses in scatter plot format. Each marker represents one of the 81 points in the discrete distribution. The open circles denote lower probability (under 0.001) combinations, which represent 59% (48/81) of the values and the filled circles represent higher probability (0.001 and above) combinations, which represent the remaining 41% of the values. Due to overlaps, not all points are visible on the graph. The scatter plots also provide insights into the relative importance of variability in each disease. For Uganda, the points fall along three distinct bands. These turn out to be linked to alternative estimates of the prevalence of malaria among pregnant women. Points in the top band all relate to a high incidence of malaria, those in the middle band to a medium incidence of malaria, and ones in the low band to a low incidence of malaria. The scatter within each band shows the comparatively smaller impact of variability in the incidence of the other three diseases.

For Kenya, for the ICER, the median value is $5.10 with a 95% confidence bound of $-6.53 to $14.27. For the benefit-cost ratio, the median is 17.45, with a 95% confidence bound of 16.46 to 18.60. For Rwanda, for the ICER, the median value is $32.58 with a 95% confidence bound of $17.54-$46.70. For the benefit-cost ratio, the median is 6.20 with a 95% confidence bound of 5.91–6.45. For Uganda, for the ICER, the median value is negative $19.46 with a 95% confidence bound of $-24.18 to $-15.42. For the benefit-cost ratio, the median is 25.36, with a 95% confidence bound of 16.88 to 33.14.

## Discussion

Our model suggests that early detection and treatment of four infections (HBV, HIV, syphilis, and malaria) through screening with the ANC panel in Africa is cost-saving in Uganda and highly cost-effective in Kenya and Rwanda. The main benefit of using the ANC panel is reducing the disease burden. The lower the pre-existing testing rate at the ANC facilities for those four infections and the higher the prevalence or incidence of the disease, the greater the benefit of complementing the current screening ANC program with the ANC panel. In Kenya, the ANC panel averted 92 DALYs per 1,000 pregnancies overall, due primarily to improvement in testing for hepatitis B and malaria, 44 and 41 DALYs, respectively. In Rwanda, the ANC panel averted 54 DALYs overall, mainly for improving testing for hepatitis B (45 DALYs). In Uganda, the ANC panel averted 258 DALYs overall, due mainly to testing for malaria infection (203 DALYs).

We estimated ICERs of $5.76 (-$6.53-$14.24), $32.62 ($17.54-$46.70), and negative $19.40 (-$24.18–$15.42) in Kenya, Rwanda, and Uganda respectively, showing the marginal cost in Kenya and Rwanda and saving in Uganda for each DALYs averted. The benefit-cost ratios show that for each $1 spent on the ANC panel, there were $17.48 ($16.46-$18.60), $6.20 ($5.91-$6.45), and $25.36 ($16.88-$33.14) economic benefits in Kenya, Rwanda, and Uganda, respectively. These results suggest that the ANC panel will be beneficial for all countries with substantial disease burdens. However, the benefits are higher in countries where the screening program is not well developed and the incidence of infections is higher. For example, in Uganda, the ANC panel was cost saving with an ICER of negative $19.40 (-$24.18–$15.42), compared to Rwanda where the ANC panel has a higher, but still very favorable ICER of $32.62 ($17.54-$46.70) per DALY averted.

Under our most likely values, our benefit-cost results found that each $1 spent on the ANC panel is associated with an economic benefit that ranges from $25 in Uganda to $6 in Rwanda. The majority of the benefit was due to early detection and treatment of hepatitis B in Kenya and Rwanda and of malaria in Uganda, the two conditions that are not usually covered by the national routine screening conducted during the first ANC visit. As a result, Uganda shows substantial savings from avoiding considerable malaria treatment. This result gives a negative net cost (i.e., cost saving) and a negative ICER in Uganda. While the ICER is not negative in the other two countries, the low value is still extremely favorable.

Some comparisons help address the affordability of the ANC panel. Based on gross costs alone, the potential budget impact of paying $4.00 per test initially appeared formidable. In Rwanda, the first year’s supply of ANC panels was donated by the manufacturer. When that initial supply was exhausted, the use of the ANC panel had to be suspended, but subsequently, additional sources of donor funding were obtained. Using a more thorough analysis using net cost, however, the budget impact is actually quite small. In Rwanda, we found that of the cost per pregnant woman of $1.77, 77% ($1.34) was averted from avoided testing and treatment, so the net healthcare cost per woman was only 23% ($0.40) of the total.

We also compared the gross and net cost to another benchmark measure, the average cost per curative case at a Second-Generation of Health Posts in Rwanda. Across eight posts, which serve the same populations, the estimated average (± standard deviation) cost of care per case from Oct 2020 to Nov 2022 was $1.34 (±$1.36) [81]. These costs per case were readily covered by a combination of patient payments and the Rwanda insurance system. The net cost per woman for the ANC panel ($0.39) represents 29% of the cost of an average visit. Thus, it is a cost that should be absorbable within the healthcare system.

The procedure in Bugesera district for syphilis testing (test once at the first ANC visit or as soon thereafter as possible) was based on affordable cost and feasibility of implementation. We acknowledge that these choices impose some penalties. Some women who test positive may not have active syphilis and, therefore, may not actually require treatment. To identify this subset, pregnant women with positive treponemal screening tests (e.g., enzyme immunoassays (EIA), chemiluminescence immunoassays (CIA), or immunoblot) could have additional quantitative nontreponemal testing to check the need for treatment, and, if confirmed, to determine the disease stage and monitor pregnant woman’s response to treatment.

On the other hand, if this validation step were included, gaps in referral and follow-up could mean that some women who should have been treated fail to receive it. Even if follow-up were perfect, however, the cost of the additional tests would likely be more than the savings from avoiding some antibiotic treatment. Based on our calculations, the potential reduction burden of syphilis due to universal testing and treatment for syphilis in pregnancy (averaging the three countries equally) is 4.04 DALYs averted per 1,000 pregnancies. The corresponding reduction for all four conditions (including HIV, HBV, and malaria) is 134.88 DALYs averted per 1,000 pregnancies across the three countries. Thus, syphilis represents only 3.0% of the DALYs averted.

In addition, the single test and associated single round of treatment will not protect against syphilis infection acquired later in pregnancy. To avoid this problem, some public health experts recommend that serological testing for syphilis be performed twice during the third trimester: at 28 weeks gestation and at delivery for pregnant women who live in communities with high rates of syphilis and for women who have been at risk for syphilis acquisition during pregnancy [82]. Our calculations suggest, however, that incorporating the possibility of reinfection during the third trimester would have minimal impact. Based on an assumption of a 3-year latency period [83] and an interval of 15 weeks for retesting, reinfection would have reduced the benefit by 0.8% of the overall gains from ANC testing.

Three types of limitations must be acknowledged with this evaluation. The first type, unknown data items, relates to data items with no known adequate sources. In Rwanda, where the ANC panel was being piloted, we had actual pilot data. There were no such data from Kenya or Uganda. We relied instead on literature, where available, and expert judgment where there were no relevant studies. While the literature generally has high internal validity, as peer-reviewed studies are usually well conducted, they tend to have low external validity. Research sites may be chosen for their unusually high rate of the chosen condition rather than their representativeness. Expert judgments are necessarily subjective. The unknown data limitations mean that the estimated model effects could be too optimistic or pessimistic, but the degree of uncertainty cannot be objectively quantified.

The second type of limitation, health system constraints, acknowledges limitations in the ability of the health system to finance and deliver recommended services. The efficacy calculations assume that the healthcare system delivers planned services completely and promptly. The ANC panel is assumed to be ordered, paid for, shipped, kept in stock, and offered to every pregnant woman in antenatal care. If a woman tests positive, she is assumed to be informed and linked to a referral system for follow-up with confirmatory testing, if needed, and appropriate treatment.

The third type of limitation concerns patients’ responses. The calculations assume that women will accept the ANC tests when offered, will obtain every requested confirmatory test, and will follow through on all the recommended referral care. The completion of all steps depends on sufficient trust in the underlying intentions, knowledge, and quality, as well as the time and money to cover the services and associated travel.

The health system and patient response limitations illustrate the saying that a chain is as strong as its weakest link. If any link were substantially broken with no alternative pathway, the health gains would be substantially reduced. Our calculations attempt to acknowledge alternative pathways. With the treatment of hepatitis B, for example, we assumed that treatment would remain efficacious even if half of the doses were missed due to the persistent effects of earlier doses [84]. This assumption is plausible given evidence showing HBV deoxyribonucleic acid (DNA) level rebounds during treatment discontinuation, but rarely does this rebound cause significant clinical flares of hepatitis, except for those with advanced fibrosis [85]. The use of this ANC panel at Second Generation of Health Posts contributes to those posts’ economic value [86].

In conclusion, using the ANC panel would allow the detection and treatment of conditions not currently covered by the national ANC screening programs. Most importantly, this is a cost-saving or highly cost-effective intervention for Kenya, Rwanda, Uganda, and similar sub-Saharan African countries. The application of this testing modality will also deliver the important benefits of improved health to the mother, the child, and the community.

## Electronic supplementary material

Below is the link to the electronic supplementary material.

**Supplemental Table S1.** The average time needed to conduct diagnostic tasks for HBV, HIV, syphilis, and malaria by the diagnostic test method

**Supplemental Material S2.** ANC panel economic evaluation

**Supplemental Table S3.** DALYs averted due to the ANC panel by country and condition per 1,000 pregmant women receiving the panel

## Data Availability

The ANC model and accompanying data are available for Rwanda, the country which has applied the ANC panel, as Supplemental Excel Workbook S2.
