# Supplementary material for: Economic analysis of a new four-panel rapid screening test in antenatal care in Kenya, Rwanda, and Uganda
Source: BMC Health Serv Res. 2023 Jul 31;23:815. doi: 10.1186/s12913-023-09775-z (PMC10391856; doi:10.1186/s12913-023-09775-z)
Supplement: Supplementary file 1 — Supplemental Table S1. The average time needed to conduct diagnostic tasks for HBV, HIV, syphilis, and malaria by the diagnostic test method [file 12913_2023_9775_MOESM1_ESM.docx]

Supplemental Table S1. The average time needed to conduct diagnostic tasks for HBV, HIV, syphilis, and malaria by the diagnostic test method^a^

| Task | Test method | Staff role | Time spent on the task (hours) |
| --- | --- | --- | --- |
| Inventory management | ANC panel | nurse | 0.05 |
| Testing procedure^b^ | ANC panel | laboratory technician | 0.50 |
| Inventory management | conventional test | nurse | 0.05 |
| Hepatitis B conventional testing procedure | conventional test | laboratory technician | 0.75 |
| HIV conventional testing procedure | conventional test | laboratory technician | 0.27 |
| Malaria conventional testing procedure | conventional test | laboratory technician | 0.27 |
| Syphilis conventional testing procedure | conventional test | laboratory technician | 0.27 |

Notes: ANC denotes antenatal care; HBV denotes hepatitis B virus

^a^ Authors’ estimate based on Dr. Sabine M Furere's interviews with staff of second generation of health posts in Rwanda.

^b^ Authors estimated that 95% of testing procedures are conducted by a laboratory technician. If no laboratory technician is on duty (the remaining 5% of testing proceudres), the authors estimated that the procedure is conducted ty a nurse.
