# Supplementary material for: Economic analysis of a new four-panel rapid screening test in antenatal care in Kenya, Rwanda, and Uganda
Source: BMC Health Serv Res. 2023 Jul 31;23:815. doi: 10.1186/s12913-023-09775-z (PMC10391856; doi:10.1186/s12913-023-09775-z)
Supplement: Supplementary file 3 — Supplemental Table S3. DALYs averted due to the ANC panel by country and condition per 1,000 pregmant women receiving the panel [file 12913_2023_9775_MOESM3_ESM.docx]

Supplemental Table S3. DALYs averted due to the ANC panel by country and condition per 1,000 pregnant women receiving the panel

| DALYs Averted | Hepatitis B | HIV/AIDS | Malaria | Syphilis | Total |
| --- | --- | --- | --- | --- | --- |
| Kenya | | | | | |
| Most favorable | 65.67 | 6.29 | 58.17 | 3.87 | 134.00 |
| Best estimate | 43.78 | 4.19 | 41.47 | 2.58 | 92.02 |
| Least favorable | 21.89 | 2.10 | 60.54 | 1.29 | 85.82 |
| Rwanda | | | | | |
| Most favorable | 46.46 | 2.75 | 4.57 | 6.25 | 60.04 |
| Best estimate | 42.97 | 1.83 | 3.02 | 4.17 | 51.99 |
| Least favorable | 39.49 | 0.92 | 1.60 | 2.08 | 44.09 |
| Uganda | | | | | |
| Most favorable | 73.56 | 0.92 | 291.87 | 8.04 | 374.39 |
| Best estimate | 49.04 | 0.62 | 203.27 | 5.36 | 258.29 |
| Least favorable | 24.52 | 0.31 | 106.68 | 2.68 | 134.19 |

Notes: DALYs denotes disability-adjusted life years; ANC denotes antenatal care.
